# Targeting glycolysis by 3-bromopyruvate improves tamoxifen cytotoxicity of breast cancer cell lines

**DOI:** 10.1186/s12885-015-1850-4

**Published:** 2015-11-03

**Authors:** Yasmin M. Attia, Hanan S. EL-Abhar, Mahmoud M. Al Marzabani, Samia A. Shouman

**Affiliations:** 1 Pharmacology Unit, Cancer Biology Department, National Cancer Institute, Cairo University, Kasr Al Eini Street, Fom El Khalig, Cairo, Egypt 11796; 2 Pharmacology and Toxicology Department, Faculty of Pharmacy, Cairo University, Kasr El-Aini St, Cairo, Egypt 11562

**Keywords:** Breast cancer, Tamoxifen, 3-bromopyruvate, Apoptosis, Angiogenesis, MMPs

## Abstract

**Background:**

Tamoxifen is the standard endocrine therapy for ER+ breast cancer; however, many women still relapse after long-term therapy. 3-Bromopyruvate, a glycolytic inhibitor, has shown high selective anti-tumor activity *in vitro*, and *in vivo*. The aim of this study was to evaluate the possible augmentation of the effect of tamoxifen via reprograming cancer cell metabolism using 3-bromopyruvate.

**Methods:**

An *in vitro* screening of antitumor activity as well as the apoptotic, anti-metastatic, and anti-angiogenic potentials of the combination therapy were carried out using different techniques on breast cancer cell lines MCF7and T47D. In addition the antitumor effect of the combined therapy was done on mice bearing tumor.

**Results:**

Our results showed modulation in apoptosis, angiogenesis and metastatic potential by either drug alone; however, their combination has surpassed that of the individual one. Combination regimen enhanced activated caspases-3, 7 and 9, as well as oxidative stress, signified by increased malondialdehyde and decreased glutathione level. Additionally, the angiogenesis and metastasis markers, including hypoxia inducing factor-1α, vascular endothelia growth factor, and metaloproteinases-2 and 9 were decreased after using the combination regimen. These results were further confirmed by the *in vivo* study, which depicted a decrease in the tumor volume and angiogenesis and an increase in oxidative stress as well.

**Conclusion:**

3-bromopyruvate could be a valuable compound when added with tamoxifen in breast cancer treatment.

## Background

Breast cancer was estimated one of the most commonly diagnosed cancers worldwide among women (11.9 %). It is the most common cause of cancer death and the most frequently diagnosed cancer in 140 out of 184 countries worldwide [1] including Egypt, where there were an estimated 49.5 cases of breast cancer per 100,000 adults in 2012 [2]. Among the different molecular subtypes of breast cancer, estrogen (ER) positive comprises ~70 % of all breast cancers cases [3].

Tamoxifen (TAM), a synthetic nonsteroidal anti-estrogen, has been used widely as the gold standard endocrine therapy for most women with ERα + breast cancer. Five years of TAM treatment reduced the risk of relapse of 10 years by 37 % in females aged 50-59 years, and by 54 % in females aged 60–69 years [4]. The antiproliferative effects of TAM may relate to its antiestrogenic effect via binding competitively to estrogen receptor, thereby blocking the mitogenic effect of estrogens [5]. TAM also induces apoptosis of cancer cell through several distinct mechanisms including its inhibition of protein kinase C and its binding to calmodulin, a protein that plays a role in DNA synthesis [6]. Although TAM is an extremely effective treatment for millions of patients with breast cancer, a significant proportion, as much as 30 % of women still relapse during or after long-term therapy [7]. Besides, some patients display de novo or acquired resistance [5].

The competency to increase response and reduce chemoresistance of cancer therapeutics via the use of the combination therapy as well uncovering underlying mechanisms of chemoresistance would be a significant advantage for cancer patients. The development of a combination therapy that increases the efficacy of TAM has been investigated in several studies, using vitamin E [8] and green tea [9]. Moreover, mounting evidence supports, that reprogramming of cellular metabolism in cancer cells is linked to failure of treatment, and drug resistance in cancer therapy [10].

The glycolysis pathway is one of the main characteristics of tumor cells, which increases dramatically with malignancy [11]. Such increased aerobic glycolysis has been observed in a variety of cancer types; hence, targeting this pathway in cancer cells provides a biochemical basis for developing new chemotherapeutic strategies. 3-Bromopyruvate (3-BP) is an inhibitor of the glycolysis process that has shown remarkable anti-tumor efficacy, documented by both *in vitro* [12] and *in vivo* [13] studies. 3-BP mediates its effect by causing cell cycle arrest, inducing apoptosis and inhibiting angiogenesis activity, which closely related to glycolysis inhibition [14]. Therefore, we hypothesized that the use of glycolytic inhibitor (3 BP) could increase TAM efficacy on MCF-7 and T47D cell lines, as well as on mice -bearing Ehrlich solid tumor as a model established in studying the effect of chemotherapy *in vivo*.

## Methods

### Drugs

Tamoxifen (TAM) and 3-BP were obtained from Sigma Aldrich Chemical Co. (St. Louis, MO, USA). Each vial of TAM contains one gm white powder. It was dissolved in DMSO to yield 50 μM then serially diluted in RPMI-1640 medium immediately before use to yield a concentration range of 10–50 μM. 3-Bromopyruvate (3-BP) was obtained in a vial containing 10 g white powder. It was dissolved in saline to yield 50 μM then serially diluted in RPMI-1640 supplemented medium immediately before use to yield a concentration range of 10–50 μM.

### Chemicals

RPMI-1640 Medium, fetal bovine serum, dimethylsulfoxide (DMSO), Ellman’s reagent [5,5-Dithio-bis-(2-nitro benzoic acid)], β-mercaptoethanol, reduced glutathione, sodium dodecyl sulfate (SDS), sodium bicarbonate, 1,1.3,3-tetramethoxypropane, trichloroacetic acid (TCA) and thiobarbituric acid were all purchased from Sigma Aldrich Chemical Co. (St. Louis, MO, USA). Triton X-100 was procured from MP Biochemical (Santa Ana, California, USA). All other chemicals and reagents were from standard analytical grade.

### Cell lines and animals

#### Cell lines

Breast carcinoma estrogen receptor positive (ER+) cell lines MCF-7 and T47D were used in this study. They were obtained frozen in liquid nitrogen (−180 °C) from the American Type Culture Collection Organization (USA). The tumor cell lines were maintained by serial sub-culturing at the National Cancer Institute, Cairo, Egypt. They were cultured in a humidified incubator at 37 °C and 5 % CO_2_ in RPMI-1640 medium supplemented with 10 % fetal bovine serum, 100 U/ml penicillin, 100 mg/ml streptomycin, and 3 mM/l glutamine. The cells were trypsinized every 3 days.

#### Animals

24 Female Swiss albino mice, weighing 22–25 g, were obtained from the National Cancer Institute, Cairo, Egypt. All of the animal handling and study procedures were approved by the research ethics committee of Faculty of Pharmacy, Cairo University, Cairo, Egypt (Permit Number: PT 661), and was conducted with the “Guide for the Care and Use of Laboratory Animals”. Animals were kept under suitable laboratory conditions of temperature and humidity. They were provided with standard chow and water and housed in plastic cages.

### *In-vitro* parameters

#### Cytotoxicity assay

To study the antitumor activity of TAM, 3BP, and their combination on breast cancer cells, sulphorhodamine-B (SRB) method as described by Skehan et al. [15] was used. In brief; cells were seeded at a density of 3 × 10^3^ cells/well in 96-well microtiter plates. They were left to attach for 24 h before incubation with drugs. Next, cells were treated with different concentrations of TAM, 3BP (10, 20, 30, 40 and 50 μM). The combination regimens was designed using IC_50_ of TAM with different concentrations (10, 20, 30, 40, 50 μM) of 3BP. For each concentration, three wells were used and incubation was continued for 48 h. A control wells containing, vehicles DMSO (1 % v/v) for TAM, and media for 3-BP were used. At the end of incubation, cells were fixed with 20 % trichloroacetic acid (TCA), stained with 0.4 % SRB dye. The optical density (O.D.) of each well was measured spectrophotometrically at 570 nm using ELISA microplate reader (TECAN sunrise™, Germany).

The mean survival fraction at each drug concentration was calculated as follows: O.D. of the treated cells/O.D. of the control cells. The IC_50_ (concentration that produce 50 % of cell growth inhibition) value of each drug was calculated using sigmoidal dose response curve-fitting models (Graph Pad Prizm software, version 5).

In all the following mechanistic experiments, we used the first concentration of 3BP that produced significant decrease of survival with IC_50_ of TAM in both cell lines. Therefore, we used in MCF-7, 20 μM of TAM, 3BP and their combination, while in T47D it was 30 μM of 3-BP and 20 μM TAM and their combination.

### Real time polymerase chain reaction (qPCR)

In order to study the effect of different treatments on angiogenesis, metastasis and apoptosis, the gene expression levels of mRNA of hexokinase (HK2), hypoxia inducing factor (HIF1-α), and metalloproteinase (MMP 2 and 9) as well as caspase 9 were assessed using q PCR. The total cellular RNA was extracted following the protocol of the RNeasy Mini Kit (Qiagen, Valencia, CA). Reverse transcription was completed using High capacity cDNA archive kit (Applied Biosystem, California, USA). Real time PCR of GAPDH, caspase 9, HK2, HIF1-α, and (MMP 2 and 9) were performed in triplicate on an ABI 7500 Fast Real-Time PCR System using the GoTaq PCR master mix (Promega, Madison, U.S.A). Fast amplification parameters were as follows: one cycle at 95 °C for 10 min, followed by 40 cycles at 95 °C for 15 s, and 60 °C for 1 min. All primers used in this study were purchased from Invitrogen (California, USA) (Table 1). Quantitative analysis of data was performed by using the∆∆ Ct method [16]. Values were normalized to GAPDH and were expressed as relative expression levels.

**Table 1:** The primer sequences of GAPDH, Caspase-9, HK-2, HIF-1 α, MMP-2 and 9 genes

Gene	Forward	Reverse
GAPDH	5-TGAAGGTCGGAGTCAACGGATTT-3	5-GCCATGGAATTTGCCATGGGTGG-3
Caspase-9	5-GGCTGTCTACGGCACAGATGG-3	5-CTGGCTCGGGGTTACTGCCAG-3
HK-2	5-CAAAGTGACAGTGGGTGTGG-3	5-GCCAGGTCCTTCACTGCTC-3
HIF-1 α	5′- CAAGAACCTACTGCTA ATGC-3	5-TTATGTATGTGGGTAGGAGATG-3
MMP-2	5-TGCCCAAGAATAGATGCTGAC-3	5-GAAAGGAGAAGAGCCTGAAGTG-3
MMP-9	5- CTTCT GCCCGGACCAAGGATAC-3	5-TTCAGGGCGAGGACCATAGAGG-3

### Assay of caspase-3 activity

#### To confirm our data different techniques as ELISA, gelatin zymography and western method were used

Caspase 3, the executioner caspase, was assessed spectrophotometrically at 450 nm in cell lysate using ELISA kit (Invitrogen, Carlsbad, CA, USA) following the manufacturer’s instructions [17]. Cells were cultured in 75 cm^3^ flasks, left till 70–80 % confluent, cells were treated with the different drug for 48 h. The treated and control cells were lysed in a RIPA lysis buffer containing protease inhibitors. Each concentration repeated two times and the experiment was carried out three independent times. The activity was calculated relative to the corresponding protein content.

### Protein concentration assay

Protein concentrations were measured in the medium and cell lysate by the method described previously by Bradford [18] using kit (Pierce, Rockford, IL, USA). The method depends on the binding of Comassie Brilliant Blue G-250 dye with protein and forming a complex which can be measured spectrophotometrically at 595 nm then the concentration was determined using a standard calibration curve.

### Assay of VEGF-A level

VEGF was determined in cell culture medium using eBioscience (San Diego, CA, USA) ELISA kit. MCF-7 and T47D cells were plated in 6 well plate with 5*10^4^ / well. After treatment with drugs, the medium was aspirated, centrifuged at 10,000 rpm for 10 min at 4 °C to remove any dead cells. The clear supernatant was used for assay following the manufacturer’s instructions [19].

### Determination of matrix metalloproteinases (MMP)-2 and 9 activities by gelatin zymography

Cells were seeded in 75 cm^3^ flasks, left for 24 h, and then treated with TAM, 3 BP, or their combination for 48 h. Cells were harvested and protein concentration of each sample was determined by Bradford method [18]. Briefly, 20 μg protein /lane was prepared in a non-reducing loading buffer consisting of 63 mM Tris–HCl pH 6.8, 10 % glycerol (v/v), 2 % sodium dodecyl sulphate (SDS) (w/v), 0.0025 % bromophenol blue (w/v), and electrophoresed on 10 % SDS-polyacrylamide gels containing 0.1 % gelatin. After electrophoresis, SDS was removed from gels by incubation with renaturation buffer (2.7 % TritonX-100) for 1 h, then incubated for 24 h at 37 °C in developing buffer (50 mM Tris–HCl, pH 7.5, 0.2 M NaCl, Triton-X 5 ml and 5 mM CaCl_2_), stained with coomassie brilliant blue and destained using destaining solution (10 % methanol, 5 % acetic acid). Enzyme-digested regions were observed as clear bands against a dark blue background. Gels were scanned using image Scanner III LabScan6.0 and the subsequent. In order to determine mean intensity of each band (mean pixel), the band densities were measured with Scion Image Beta 4.0.2 (Scion Co., Frederick, MD, U.S.A.) software. For the quantitative analysis, each of the bands was compared with β-Actin taken as a control.

### Western blot

Cells were washed twice with PBS and lysed in cell lysis buffer (150 mM NaCl,10 mM Tris, 0.2 %TritonX-100, 0.3 %nonylphenoxy-polyethoxyethanol-40, 0.2 %mM Na_3_VO_4_, protease inhibitor cocktail). The cell lysates were centrifuged and the protein concentration was measured as previously mentioned. Each sample was separated by electrophoresis using 8 % SDS-PAGE gel and analyzed by Western blotting using the following antibodies: primary rabbit anti-human MMP-9 (Novusbio, Colorado, USA), and β-HK2 (Cell signaling, Beverly, Massachusetts, USA), as well as primary mouse anti-human HIF-1α (eBioscience, CA, USA), MMP-2 (Invitrogen, CA, USA), caspase-7 (Novusbio, Colorado, USA), and β-Actin (Sigma-Aldrich Chemical Co., USA). Horseradish peroxidase linked to the corresponding secondary antibody was used at 1:5000 dilution. The membrane was visualized by exposure to Kodak XAR film. For the quantitative analysis, the mean intensity of each band (mean pixel), was compared with β-Actin band using with Scion Image Beta 4.0.2 (Scion Co., MD, U.S.A.) software.

### Oxidative stress markers (reduced glutathione and lipid peroxide)

In order to explore the role of oxidative stress in drug - induced cytotoxicity, levels of lipid peroxide and reduced glutathione (rGSH were determined. Glutathione content was determined according to the method of Ellman [20]. The treated and control cells were collected in phosphate buffer, protein was precipitated with trichloroacetic acid (TCA) and centrifuged. The supernatant was treated with Ellman’s reagent, the developed color was measured spectrophotometrically at 405 nm using a spectrophotometer (Spectronic, Milton Ray Co., USA). Lipid peroxidation products were quantified by measuring malonaldialdehyde (MDA) level to the method described by Draper and Hadley [21]. Treated and control cells were mixed well with of 20 % (w/v) trichloroacetic acid (TCA) containing 0.8 % (w/v) thiobarbituric acid (TBA), incubated in a boiling water bath for 1 h. The absorbance of the supernatant was determined at 535 nm using a spectrophotometer (Spectronic, Milton Ray Co., USA). The concentrations were calculated using MDA standard calibration curve by preparing a serial dilutions of 1,1,3,3- tetraethoxypropane.

### *In-vivo* parameters

#### Assessment of the antitumor activity in mice-bearing solid Ehrlich carcinoma (EAC)

Ehrlich carcinoma (EAC)-cells (2 × 10^6^) were transplanted subcutaneously in the right thigh of the lower limb mice. 24 Mice with a palpable tumor mass (approximate 100 mm^3^), which developed within 7 days after implementation, were divided randomly and blindly into 4 groups each 6 animals. Group one injected i.p with 5 mg/kg TAM, group two injected with 3-BP (10 mg/kg), group three treated with their combination and control group received saline. Treatment continued twice/weekly for 3 weeks. The change in tumor volume was measured using venire caliber and calculated by the following formula according to Osman et al. [22].

$$ \mathrm{Tumor}\kern0.5em \mathrm{volume}\kern0.5em \mathrm{m}{\mathrm{m}}^3=0.52\kern0.5em {A}^2\times \kern0.5em \mathrm{B} $$

Where A and B denote the minor and major tumor axis, respectively.

### Reduced glutathione (rGSH) and MDA contents in solid tumor tissue

Twenty four hours after the last treatment, animals were anesthetized with sodium pentobarbital 100 mg/kg i.p, then cervical dislocation was done with high degree of proficiency to anesthetized animals according to Euthanasia guidelines. Tumors were quickly excised, washed with saline, blotted with a piece of filter paper, and homogenized using a Branson sonifier (250, VWR Scientific, Danbury, Connecticut, USA). The homogenates were centrifuged at 800 g for 5 min at 4 C° to separate the nuclear debris, then supernatant was again centrifuged at 10,500 g for 20 min at 4 C°. Levels of glutathione and MDA were determined as previously described.

### Immunohistochemical staining (IHC) of VEGF

Representative tissue samples were fixed in 10 % neutral phosphate-buffered formalin, embedded in paraffin, and sectioned at 5 μm thickness. Sections were incubated with monoclonal mouse anti-VEGF antibody (Sigma Aldrich Chemical Co., USA) as a primary antibody at a dilution of 1:150 overnight at 4 °C then rinsed three times. Sections were incubated with polymer horseradish peroxidase HRP secondary antibody (Sigma Aldrich Chemical Co., USA) for 1 h. Immuno-reactivity was detected by the standard avidin–biotin immunoperoxidase method. Counterstaining with Meyer’s hematoxylin was then performed for 5 min. Thereafter, they were evaluated under light microscope (Olympus, Japan) and analyzed with Scion Image Beta 4.0.2 (Scion Co., Frederick, MD, U.S.A.) software.

### Statistical analysis

All data were expressed as mean ± S.D. The difference between the treated samples and the untreated controls was analyzed by one way ANOVA followed by Tukey multiple comparison test in which *p* < 0.05 was considered as significant. To test for interaction between individual treatments when given in combination, a factorial design test is used. All statistical analysis was performed using GraphPad In Stat, version 5.0 (GraphPad, San Diego, California, USA). Compusyn software was used to determine the interaction between the two drugs in the combination. Statistical significance was set at *p* < 0.05.

## Results

### *In vitro*

#### 3-BP enhances cytotoxicity of TAM on MCF7 and T47D cells

**Fig. 1:**
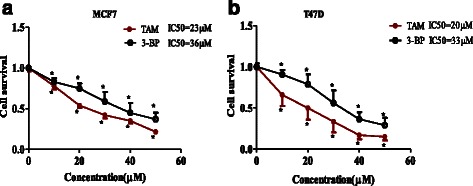
Cytotoxicity of TAM and 3-BP on MCF7 and T47D breast cancer cell lines after 48 h. Surviving fraction and I.C_50_ of MCF-7 (**a**) and T47D (**b**), cells treated with TAM and 3-BP after 48 h. Results are expressed as the mean ± SD of 5 independent experiments performed in triplicate. ^*^ Significantly different from control at *P* < 0.05

**Fig. 2:**
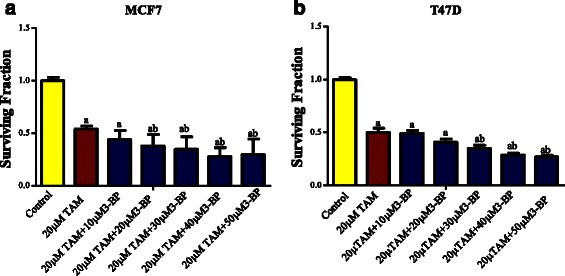
Effect of addition of 3-BP on the cytotoxicity of 20 μM TAM on MCF-7 and T47D cell lines. Cells were treated with different concentrations of 3-BP and 20 μM TAM (**a**, **b**, respectively). Results are expressed as the mean ± SD of 5 independent experiments performed in triplicate. The statistical significance of the results was analyzed by one way ANOVA using Tukey multiple comparison test using one way analysis of variance (ANOVA). “^a^” Significantly different from its control and “^b^” from 20 μM TAM at *P* < 0.05

Figure 1 showed that treatment of MCF7 [A] and T47D [B] cells with various concentrations (10–50 μM) of TAM or 3-BP for 48 h caused a concentration dependent decrease in cell survival. The IC_50_ of TAM was 20 and 23 μM, while that of 3-BP was 36 and 33 μM in MCF7 and T47D, respectively. Addition of 20 μM of 3BP increased significantly cytotoxicity of 20 μM TAM in MCF-7 cells, while, T47D cells required 30 μM of 3BP to produce significant increase in cell death compared to TAM alone (Fig. 2a and b).

#### 3-BP synergizes oxidative stress and activates apoptotic machinery of TAM on MCF7 and T47D cells

**Fig. 3:**
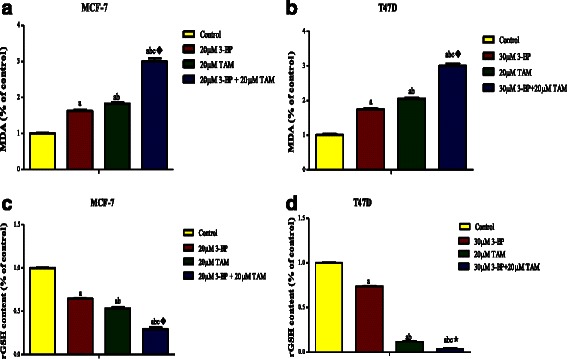
Oxidative stress markers following treatment with TAM, 3-BP and their combination. Effect of different regimen on lipid peroxidation in MCF-7 (**a**) and T47D (**b**). Figure (**c**) and (**d**) show the content of reduced glutathione (rGSH) in MCF-7 and T47D, respectively after 48 h treatment with 3-BP, TAM and their combination. Results are expressed as means ± SD of 2 independent experiments performed in duplicates. Statistical significance of results was analyzed by one way ANOVA using Tukey’s multiple comparison test. “^a^” Significantly different from control, “^b^” from 3-BP and “^c^” from TAM at *P* ≤ 0.05. ^♦^ means synergistic and * means potentiating interaction when TAM and 3-BP where combined using factorial design

**Fig. 4:**
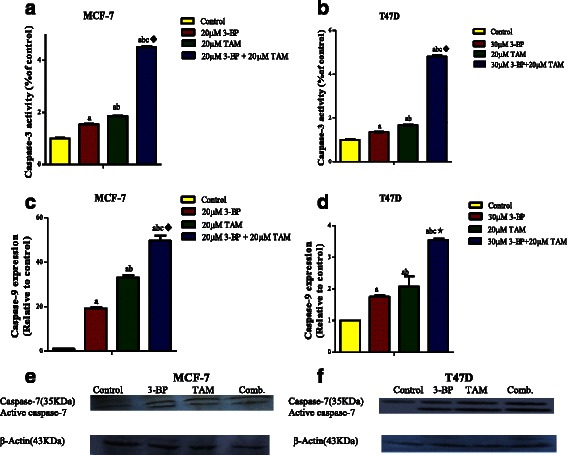
Effect of 48 h treatment with 3-BP, TAM and their combination on apoptosis markers. Caspase-3 activity in MCF-7 cells (**a**) and T47D cells (**b**). Expression of Caspase −9 gene using qPCR in MCF-7 (**c**) and T47D (**d**). Caspase 7 protein level was done by western in MCF-7 (**e**) and T47D (**f**). Results are expressed as means ± SD of 2 independent experiments performed in duplicates. Significance was determined with one way ANOVA using Tukey’s multiple comparison test. “^a^” Significantly different from control, “^b^” rom 3-BP and “^c^” from TAM at P ≤ 0.05. ^♦^ means synergistic and * potentiation interaction when TAM and 3-BP where combined using factorial design

Both TAM and 3-BP increased significantly the MDA level (Fig. 3a, b), but leveled off the rGSH content (Fig. 3c, d) significantly in the two breast cancer cell lines. The addition of 3-BP to TAM caused synergistic effect on the oxidative stress (lipid peroxidation) in both cell lines and a synergistic effect on glutathione content in MCF-7 but in T47D cells, the interaction was potentiation. Treatment of breast cell lines with TAM, 3-BP and their combination has switched on the apoptotic activity assessed as caspases 3, 7 and 9. The effect of the different treatment regimens had activated caspase-3 (Fig. 4a, b), with the 3-BP showing the least effect and the combined treatment showing the highest action with synergistic interaction. The same pattern was mirrored in the 2 cell lines. The same effect was observed on the expression of caspase-9 (Fig. 4c, d) but the interaction was synergistic on MCF-7 and potentiation on T47D cells. Additionally, the three treatments succeeded to cleave caspase-7 as shown in (Fig. 4e, f) using western blot.

#### Combined treatment of TAM and 3BP inhibits VEGF-A, HIF-1α, HK-2 and metalloproteinases 2, 9

**Fig. 5:**
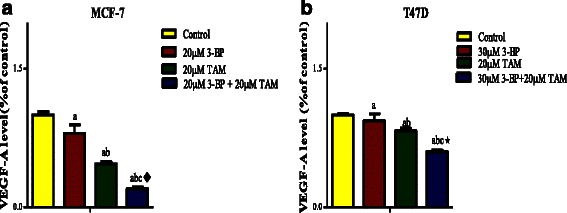
Levels of VEGF in breast cancer cell lines following treatment with 3-BP, TAM and their combination. Effect of TAM, 3-BP and their combination on level of VEGF-A in the MCF-7 (**a**) and T47D (**b**) cells media. Results are expressed as means ± SD of 2 independent experiments performed in duplicates. Significance was determined with one way ANOVA using Tukey’s multiple comparison test. “^a^” Significantly different from control, “^b^” rom 3-BP and“^c^” from TAM at P ≤ 0.05. ^♦^ means synergistic and * potentiation interaction when TAM and 3-BP where combined using factorial design

**Fig. 6:**
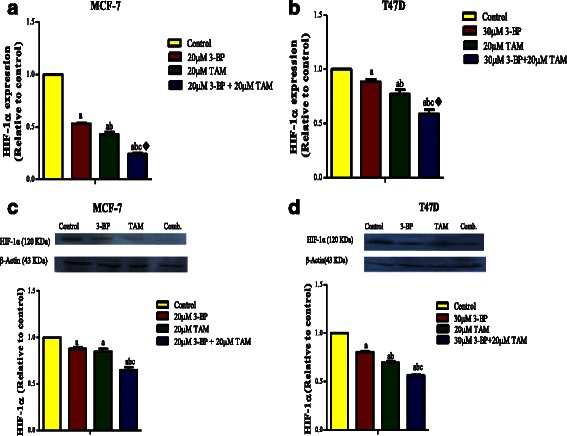
Effect of TAM, 3-BP and their combination on the level HIF-1α. The expression level of HIF-1α in MCF-7 and T47D cells (**a**, **b**). The effect of different treatments on the protein level (**c**, **d**). Results are expressed as means ± SD of 2 independent experiments performed in duplicates for qPCR experiment. The results for western blot are expressed as means ± SD of 3 independent experiments. Significance was done by one way ANOVA using Tukey’s multiple comparison test. “^a^” Significantly different from control, “^b^” from 3-BP and “^c^” from TAM at P ≤ 0.05. ^♦^ means synergistic and * potentiation interaction when TAM and 3-BP where combined using factorial design

**Fig. 7:**
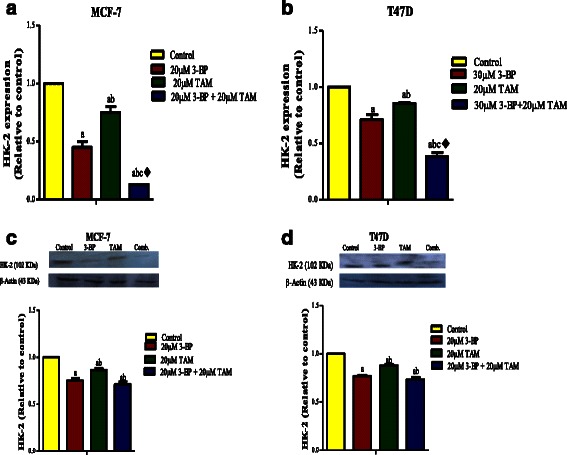
Effect of TAM, 3-BP and their combination on the level Hexokinaes-2 (HK-2). HK-2 gene expression of 3-BP, TAM and the combination regimen (**a**, **b**). The effect of different treatments on the HK-2 protein level. (**c**, **d**). Results are expressed as means ± SD of 2 independent experiments performed in duplicates for qPCR experiment and for western blot the results are expressed as means ± SD of 3 independent experiments. Significance was done by one way ANOVA using Tukey’s multiple comparison test. “^a^” Significantly different from control, “^b^” from 3-BP and “^c^” from TAM at *P* ≤ 0.05. ^♦^ means synergistic and * potentiation interaction when TAM and 3-BP where combined using factorial design

**Fig. 8:**
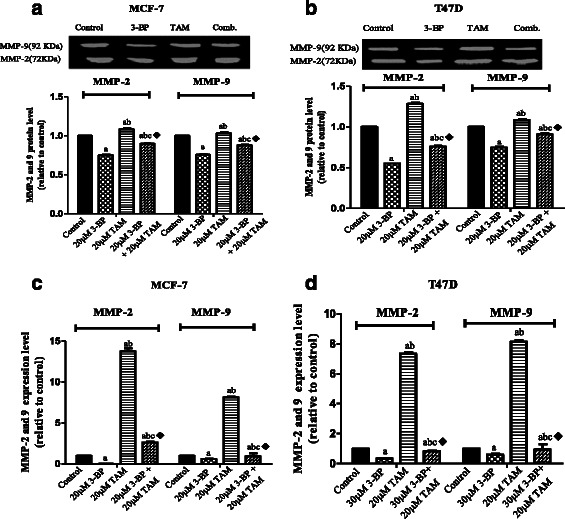
Effect of TAM, 3-BP and their combination on the extracellular level and the expression of Metastasis markers. After adding 3-BP to TAM succeeded to decrease the extracellular level of MMP-2 and 9 using gelatin zymography in MCF-7 cells (**a**) and T47D (**b**) cells. The analysis was done by image software. The effect of this combination on the secreted MMPs was reflected on their genes expression using qPCR in MCF-7 (**c**) and T47D (**d**). Results are expressed as means ± SD of 2 independent experiments for zymography but for qPCR results are expressed as means ± SD of 2 independent experiments performed in duplicates. Significance was determined with one way ANOVA using Tukey’s multiple comparison test.“^a^” Significantly different from control, “^b^” from 3-BP and “^c^” from TAM at *P* ≤ 0.05. ^♦^Significant interaction (antagonism) when TAM and 3-BP where combined using factorial design

**Fig. 9:**
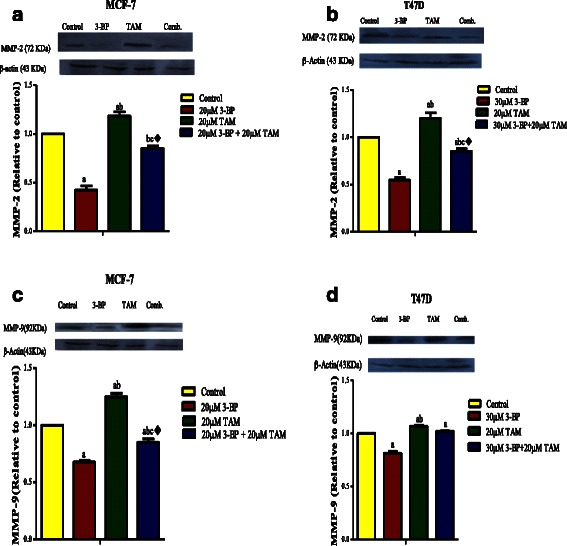
Effect of TAM, 3-BP and their combination on the protein level of the Metastasis markers. The results of zymography and qPCR were confirmed also by western technique for MMP-2 and 9 in the cells of MCF-7 (**a**, **c**) and T47D (**b**, **d**). Results are expressed as means ± SD of 2 independent experiments western. The analysis was done by image software. Significance was determined with one way ANOVA using Tukey’s multiple comparison test.“^a^” Significantly different from control, “^b^” from 3-BP and “^c^” from TAM at P ≤ 0.05. ^♦^Significant interaction (antagonism) when TAM and 3-BP where combined using factorial design

As depicted in Fig. 5a, b, VEGF-A activity was inhibited by the combined regimen showing the best effect with synergistic interaction on MCF-7 and potentiating interaction on T47D. Regarding the effect on the HIF-1α expression (Fig. 6a, b), TAM and/or 3-BP showed the same previous pattern with a more pronounced effect on the MCF-7 cell line. Nevertheless, these results were not reflected exactly on the HIF-1α protein content assessed by the western blot technique (Fig. 6c, d) as the interaction was synergistic in the expression level but it was potentiation one in protein level. The expression and the protein level of HK2 were presented in Fig. 7a-d. As expected the inhibitory effect of 3-BP on the HK2 surpassed that of TAM alone in the 2 breast cell lines studied herein. Despite the combination effect added a further inhibition in the HK2 expression as compared to the 3-BP alone with synergistic interaction, however, this effect was lost in the protein verification (Fig. 7c, d). TAM increased MMP 2 and 9. Surprisingly, 3-BP caused a sharp decline in the MMPs in the two breast cell lines to reach even a lower level below the untreated control group. The combination regimen succeeded to lower the TAM effect on the secreted MMP 2 and 9 (Fig. 8a, b); the same pattern was observed by the q-PCR technique (Fig. 8c, d) and the Western blot assay (Fig. 9a-d) with antagonistic interaction.

### *In vivo*

#### 3-BP enhances the antitumor effect, increases oxidative stress and inhibits VEGF of TAM *in vivo*

**Fig. 10:**
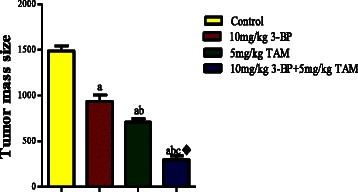
Tumor volume of solid Erlich carcinoma-bearing mice after treatment with 3-BP, TAM or their combination. The tumor volume was markedly reduced in mice treated 3-BP (10 mg/kg), TAM (5 mg/kg); however the best result was observed in group treated with combination of both drugs. Results are expressed as means ± SD of tumor volume from 6 mice. Results are analyzed by one way ANOVA using Tukey’s multiple comparison test. “^a^” Significantly different from control,“^b^” from 3-BP and “^c^” from TAM at *P* < 0.05. ^♦^Significant interaction when TAM and 3-BP where combined using factorial design

**Fig. 11:**
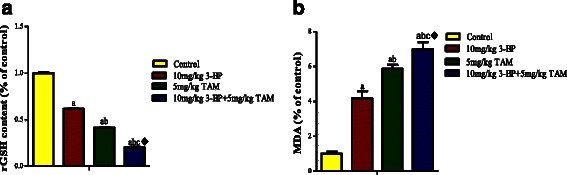
Effect of TAM, 3-BP and their combination on oxidative stress in vivo. **a** shows the level of the Glutathione level and **b** shows the lipid peroxidation following treatment with 3-BP and TAM and their combination. Results are expressed as means ± SD of tumor volume from 6 mice. Results are analyzed by one way ANOVA using Tukey’s multiple comparison test. “^a^” Significantly different from control,“^b^” significantly different from 3-BP and “^c^”significantly different from TAM at *P* < 0.05. ^♦^Significant interaction when TAM and 3-BP where combined using factorial design

**Fig. 12:**
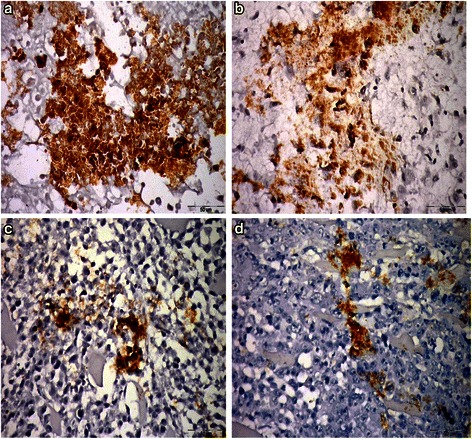
VEGF expression using immunohistochemsitry. **a**: Paraffin section photograph of mouse solid tumor (control group) showing the increased VEGF expression in most tumor cells, the large capillaries and hyperchromatic cells were present (4/5 of the field). **b**: Paraffin section photograph of mouse solid tumor (group treated with 10 mg/kg3-BP) showing the VEGF expression in most tumor cells absent the apoptotic cells (3/5 of the field). **c**: Paraffin section photograph of mouse solid tumor (group treated with 5 mg/kg) showing the VEGF expression in some tumor cells, absent in mitotic and apoptotic cell, large conjugated blood vessels were observed. (1/5 of the field). **d**: Paraffin section photograph of mouse solid tumor (group treated with 3-BP and TAM) showing the VEGF expression in some tumor cells (1/5 of the field)

The results of *in vitro* are also documented *in vivo*, the volume of *Ehrlich* tumor was decreased by 52 and 37 % in individually treated TAM or 3-BP respectively; however, the combination regimen caused a further decrease reaching 80 % as compared to the control untreated group (Fig. 10). An increase in MDA and decrease rGSH with synergistic interaction in the combination using factorial design was also observed (Fig. 11a, b). Moreover, as presented in Fig. 12a-d, all the treatment regimens lowered the level of VEGF expression to different extent when compared to the control group. Moreover, in the combination treated group the expression was even less than either treatment alone.

## Discussion

Breast cancer (BC) is the most commonly diagnosed cancer and the leading cause of cancer-related deaths among females worldwide [1]. ER status is the most important and primary determinant of treatment options through targeting ER functions by TAM or synthesis by aromatase inhibitors [23]. TAM is the first endocrine therapy; it acts as an antagonist for estrogen receptors in pre and postmenopausal breast cancer by controlling the binding of estradiol to the ER and forms a TAM-ER complex which then binds to DNA. This leads to the failure of transcriptional activation and growth inhibition in estrogen-dependent cells [5].

Our data showed either TAM or 3BP alone or in combination inhibited the survival of breast cancer cell lines as well as in mice bearing EAC tumor. The combination regimen enhanced significantly the growth inhibition both *in vitro* and *in vivo*. TAM was reported as effective anticancer against many types of cancer other than breast including hepatocellular carcinoma, lung cancer [24, 25] and colon cancer [26] cell lines. The *in vitro* findings were further elucidated in an *in vivo* model of EAC bearing mice. The present study showed that TAM and 3-BP can reduce the volume of solid tumor in mice bearing tumor. Several studies have also documented the antitumor effect of TAM and 3BP *in vivo* [13, 50]. Moreover, the combination of both drugs reduced the tumor significantly from TAM or 3-BP treated groups. It increases the level of p53 which is responsible for activation of many genes to induce apoptosis [27]. In addition, TAM causes induction of c-Myc, activation of members of mitogen-activated protein kinase (MAPK) family as well as increased accumulation of ceramide which serves as a second messenger in cell survival [28]. Moreover, 3-Bromopyruvate (3-BP) is a promising glycolytic inhibitor, in this study; it increases significantly the cytotoxicity of TAM. 3BP was found to have anticancer effects on many types of cancer including; leukemia [29], breast cancer cell line and hepatocellular carcinoma [30]. This may be related to the ability of 3BP to act as multi-targeted inhibitor of glycolytic pathway and mitochondria. It covalently binds to the glycolytic enzymes; hexokinase-2 [31], Glyceraldehyde-3-phosphate dehydrogenase [32] and mitochondrial; succinate dehydrogenase [33], in addition, to the endoplasmic reticulum [27] and the lysosomes [32] resulting in severe depletion in ATP and cancer death [34].

The antitumor effects of TAM observed in this study, was accompanied by significant increase in ROS and activation of different caspases at both m RNA and protein levels resulting in induction of apoptosis. Additionally, both the individual drug and combination treated mice showed increase in the oxidative stress markers *in vivo*. TAM increases mitochondria oxidative stress markers *in vitro* and *in vivo* [35] and induces collapse of mitochondrial transmembrane potential [36] that triggers release of cytochrome c from mitochondria which activates pro-caspase-9,7 and 3 leading to apoptosis [37]. In addition, oxidative stress increases intracellular Ca^2+^concentrations leading to leak in the plasma membrane [38] and activation of endonucleases which degrade DNA and, ultimately, contribute to cell death [39].

In our study the apoptotic effect of TAM is enhanced upon its combination with 3BP in both breast cancer cell lines compared to each drug individually. 3-Bromopyruvate, as a member of the mitocans, it exerts its pro-apoptotic mechanism on cells via disruption the mitochondria membrane potential causing the generation of mitochondrial ROS [40]. One of the major consequences of the disruption of the mitochondrial membrane potential by reactive oxygen species (ROS) is the release of the cytochrome c [41] in the cytosol and initiation of the caspase cascade by activating pro-caspase-9. Mature caspase-9 then activates the executioner caspases including caspase-3which is a point of no return in apoptosis. Caspase-3 then cleaves a variety of vital biological macromolecules [42].

As the tumor cells proliferate, they are subjected to hypoxia and undergo biological changes to adapt themselves to the hypoxic conditions. HIF-1α mediates many of the changes, [43] which regulate many genes in glycolytic pathway as hexokinase-2 (HK2) which plays a key step in glycolysis and angiogenesis as (VEGF) [44]. HIF-1α has been reported to be over expressed in various malignant tumors and cancer cell lines [45]. In our study TAM, 3BP as well as their combination inhibited the expression and the protein content of HIF-1 α with concomitant inhibition of HK and VEGF. However, the expression levels of both HIF-1 α and HK of the combined treatment in both cell lines showed synergistic effect which did not appear in the protein level carried out by Western blotting. Such difference between mRNA expression and protein level may be due to several biological and methodological constraints that play a role when comparing mRNA to protein levels [46]. The most prominently influences the correlation between mRNA and protein are the translation efficiency or protein half-life. Individual protein half-lives range from several seconds to tens of hours [47], a more than 1000-fold range. Hence protein turnover is probably influencing the correlation between mRNA and protein abundances to a greater degree. Minor effects are attributed translation initiation, start codon, stop codon and stop codon context [48] and [49]. HIF-1α plays an important role in tumor angiogenesis and high levels of HIF-1 α can predict an early relapse and metastatic disease [50]. Additionally, HIF-1α overexpression is associated with increased VEGF expression in many different types of cancer such as; breast cancer, colon cancer and hepatocellular carcinoma [51]. However, the role of estrogens and tamoxifen and in the clinic and HIF-1 α modulation in breast cancer is unclear [52]. 3-BP was reported to decrease the level of HIF-1 α [53] and covalently binds to HK-2, causing its dissociation from VDAC [31]. 3BP causes cancer cell death by rapid depletion of ATP and suppresses tumor growth in animal model [54]. The combined treatment produced significant decrease in VEGF compared to either drug alone, the effect was synergistic in MCF7, while it was additive in T47D. This difference in drug interaction between the two types could be attributed to the aggressive nature of T47D compared to MCF7. According to their biological functions, the proteins involved in cell growth stimulation, anti-apoptosis mechanisms and carcinogenesis are more strongly expressed in T47D than in MCF7 [55]. Moreover, *in vivo* the expression level of VEGF was decreased in mice treated combination regimen significantly compared to TAM or 3-BP treated groups.

Metalloproteinase degrade extracellular matrix components enabling tumor cell invasion and metastasis. It was found that estradiol and TAM regulate MMP-2, MMP-9 and extracellular endostatin in ER + PR + human breast cancer cells and *in vivo* [56, 57]. A significant increase of intracellular and secreted protein levels upon TAM exposure whereas, estradiol induced a significant decrease [56]. The authors suggested a possible role of MMP in regulation the bioavailability of a variety of biologically active molecules such as anti-angiogenic fragments, which may be beneficial for the host [56, 57]. Surprisingly in this study, 3-BP caused a sharp decline in the MMPs in the two breast cancer cell lines, contrary to TAM which caused increased levels of both enzymes. However, the combination regimen succeeded to lower the increased effect of TAM by all tested techniques (Fig. 7a-h) with antagonistic interaction. Therefore TAM and 3BP the ability to modulate MMP-2/MMP-9 activity and VEGF levels in human breast cancer *in vitro*.

## Conclusion

3-BP is a promising antitumor, it improved antitumor effect of TAM on breast cancer cell lines and in mice bearing-Ehrlish carcinoma. The combination regimen increases the antitumor effect via activation of apoptotic machinery, decreases angiogenesis markers HIF, HK2 and VEGF. Moreover 3BP modulates MMPs 2 and 9 which makes its combination with TAM promising treatment to be applied clinically.

## Abbreviations

3-BP: 3-Bromopyruvate
BC: Breast cancer
EAC: Ehrlish ascites carcinoma
ER+: Estrogen receptor positive
HIF: Hypoxia inducing factor
HK2: Hexokinase2
MDA: Malondialdehyde
MMPs: metalloproteinases
rGSH: Reduced glutathione
ROS: Reactive Oxygen Species
TAM: Tamoxifen
VEGF: Vascular endothelial growth factor
